# Veterinary Medical Association of New Jersey

**Published:** 1891-05

**Authors:** W. H. Cooper

**Affiliations:** Secretary


*Veterinary Medical Association of New Jersey.*—The seventh annual meeting of the Veterinary Medical Association of New Jersey was held at the State Street House, Trenton, N. J., on Thursday April 9, 1891.

The meeting was called to order, the President, Dr. J. W. Hawk, of Newark, in the chair. The secretary called the roll and the following members were present: Drs. J. W. Hawk, Newark; H. Bradshaw, Trenton; A. Brown, Windsor; S. S. Cole, Millville; J. C. Dustan, Morristown; J. Hurley, Hopewell; B. F. King, Little Silver; S. Lockwood, Woodbridge; W. H. Rowland, Newark; W. Runge, Newark; J. Huilson, Jersey City; R. O. Hasbrouck, Passaic; W. W. Curry, Jersey City; J. Gerth, Jr., Newark; T. De Clyne, New Durham; J. M. Everitt, Hacketstown; M. M. Stage, Dover; A. W. Axford, Naughright; and W. H. Cooper, Trenton.

The minutes of the last regular meeting of the special meeting were read and approved. President J. W. Hawk, made an address of which the following is a synopsis:

On legislative bill, and registration; no contagious diseases in the State, not a case of pleuro pneumonia within our borders; little trouble among horses at the present time; the Bureau of Animal Industry work, giving the history of the work done in New Jersey for the last nineteen months, the number of animals killed and the cost of the same, the great work done and being done by the Bureau; and sound advice to the association for its future welfare. No unfinished business. Secretary Cooper read his yearly report which was accepted and ordered on minutes. Treasurer King read his yearly report, accepted and ordered on minutes.

The chair appointed Dr. Brown to fill vacancy in Board of Trustees. The chair declared an intermission for the Board of Censors and Trustees to make their report. President recalled to order. Dr. Dustan, chairman (Pro Tem.), made the following report. On legislative bill, also on constitution and by-laws, and advised the association to prosecute unregistered veterinarians. Moved report be received, seconded and carried. The secretary read report in full. Dr. Huilson read an essay, also Dr. King. Discussion of papers, Drs. Hawk, King, Dustan, Runge, and others.

Dr. E. Britton, Long Branch, N. J., was proposed for membership, laid over according to by-laws until next meeting.

On election of officers for the ensuing year the following were chosen: President, Dr. R. R. Letts, Hoboken; First Vice-President, Dr. J. C. Dustan, Morristown; Second Vice-President, Dr. W. Runge, Newark; Secretary, Dr. W. H. Cooper, Trenton; Treasurer, Dr. B. F. King, Little Silver; Board of Censors, Dr. J. W. Hawk, chairman; Dr. W. B. E. Miller, Camden; Dr. J. Huilson, Jersey City; Dr. A. Brown, Windsor; Dr. W. W. Curry, Jersey City.

The chair appointed Dr. Dustan to escort the newly elected President, Dr. Letts, to the chair. Dr. Hawk welcomed Dr. Letts to the chair with a short address.

Dr. R. R. Letts in taking the chair made a short address, in which he spoke very feelingly of the honor bestowed upon him, and thanked the members for their esteem in choosing one of their youngest members to be their president; he hoped that he would be able to leave the chair with honor as the one who has just vacated the highest offlce of this association.

President Letts appointed Dr. Hawk to escort the other newly elected officers and introduce them to the association, which was followed by short addresses from each.

After considerable business was transacted, adjourned for the Banquet. Meeting recalled to order.

John O. Pitney, of Newark, was elected the counselor of the association.

The President appointed the following as Delegates to the different associations :

To United States Veterinary Medical Association, J. C. Dustan and W. W. Curry; New York Veterinary Medical Association, T. De Clyne and A. Oxford; Pennsylvania Veterinary Medical Association, J. Huilson and W.B. E. Miller; Maryland Veterinary Medical Association, W. Kunge and J. Gerth, Jr. Next meeting will be held at Newark, second Thursday in August, 1891. Adjourned.

W. H. Cooper, *Secretary.*

